# Crowding-out effect of tobacco consumption in Indonesia

**DOI:** 10.1136/tc-2022-057843

**Published:** 2024-01-22

**Authors:** Arya Swarnata, Fariza Zahra Kamilah, I Dewa Gede Karma Wisana, Yurdhina Meilissa, Gita Kusnadi

**Affiliations:** 1 Center for Indonesia's Strategic Development Initiatives, Jakarta Pusat, Indonesia; 2 Lembaga Demografi, Universitas Indonesia Faculty of Economics and Business, Depok, West Java, Indonesia

**Keywords:** Economics, Low/Middle income country, Price, Socioeconomic status

## Abstract

**Background:**

Tobacco consumption is pervasive in Indonesia, with 6 out of 10 households in the country consuming tobacco. Smoking households, on average, divert a significant share (10.7%) of their monthly budget on tobacco products, which is higher than spending on staples, meat or vegetables. Nevertheless, evidence of the causal link between tobacco expenditure and spending on other commodities in Indonesia is limited.

**Objective:**

This study aims to estimate the crowding-out effects of tobacco spending on the expenditure of other goods and services in Indonesia.

**Method:**

This research estimates the conditional Engel curve with three-stage least square regression, where the instrumental variable technique is applied to address the simultaneity of tobacco and total non-tobacco spending. The study employs a large-scale household budget survey from the Indonesian socioeconomic survey (Susenas) from 2017 to 2019, comprising over 900 000 households.

**Finding:**

Tobacco spending crowds out the share of a household’s budget allocated for food, such as spending on staples, meat, dairy, vegetables and fruits. Moreover, tobacco spending also reduces the share of expenditure spent on non-food commodities, such as clothing, housing, utilities, durable and non-durable goods, education, healthcare and entertainment, although its effect is not as large as the crowding out on food. The analysis shows that the crowding-out effects of tobacco are observed across low-income, middle-income and high-income households. In addition, the simulation suggests that reducing tobacco expenditure will increase household spending on essential needs.

**WHAT IS ALREADY KNOWN ON THIS TOPIC**

- Tobacco expenditure accounts for a significant share of household spending in Indonesia, with over one-tenth of the monthly budget diverted to buy cigarettes and other tobacco products.
- Smoking households in Indonesia have relatively poorer diets and lower nutritional intake compared with non-smoking families.

**WHAT THIS STUDY ADDS**

- This study provides causal evidence that tobacco spending crowds out spending on foods and non-food commodities.
- This study shows that tobacco expenditure is associated with increased spending on ready-made foods and beverages, which include caffeine and sweetened drinks.
- This study simulates that a substantial reduction in tobacco expenditure will potentially increase household spending on essential needs.

**HOW THIS STUDY MIGHT AFFECT RESEARCH, PRACTICE OR POLICY**

- This study’s findings highlight the need for a more effective tobacco control policy supporting a reduction in tobacco consumption and tobacco expenditure in Indonesia.

## Introduction

Indonesia’s tobacco consumption remains steadily high. According to the Global Adult Tobacco Survey, the prevalence of tobacco use among Indonesia’s adult populations has experienced insignificant changes as it only marginally dropped from 36.1% in 2011 to 34.5% in 2021.^1^ This means that after accounting for the population growth, the number of smokers in the country has increased over the years. In fact, it has been estimated that over 8.8 million more Indonesians consumed tobacco in 2021 than a decade earlier.^2^

Because of this pervasiveness of tobacco use, most Indonesian families are smoking households, where at least one of the members is a smoker. The National Socioeconomic Survey (2017–2019) showed that 6 out of 10 households in Indonesia reported spending on cigarette products. The survey further revealed that smoking households diverted significant resources to tobacco, where on average, around 10.7% of the households’ monthly budget was spent on buying cigarette products—higher than the spending for staples (10.4%), fruits and vegetables (6.7%), and meat (6.5%).

Although smoking in Indonesia is only prevalent among adult men, it might adversely affect the welfare of all household members, including non-smokers who live and share resources with smokers. This is because individuals often pool their income into a single household budget and share resources for daily consumption, such as spending on groceries and utilities. Therefore, the higher the tobacco spending, the less budget is available for other commodities. This is particularly the case when smokers in the household hold significant power in determining budget allocation, for example, the household head or the breadwinner. Studies have shown that male household heads in Indonesia have complete autonomy to spend household income on cigarettes before passing it on to wives, who are typically responsible for allocating the household budget for daily shopping.^3^

Owing to the fact that smoking households in Indonesia divert a significant share of their budget to tobacco consumption, one would ask whether it crowds out spending on food and other non-tobacco commodities. Previous studies have indicated that poor smoking households in Indonesia divert a substantial portion of an already limited resource to tobacco, reducing their dietary quantity and quality and consequently contributing to a lower nutritional intake among the smoking families.^4–6^ Over the long run, this crowding-out effect might adversely affect human capital investment, as studies have shown that children living in smoking families in Indonesia are exposed to a higher risk of stunting.^7^

The crowding-out effect of tobacco spending has been widely documented in other countries, such as India, Pakistan, Cambodia, Bangladesh, Turkey, South Africa, Zambia, Serbia and Vietnam.^8–16^ These studies show that tobacco expenditure crowds out spending on food and non-food commodities, such as clothing, housing, durable goods, education and healthcare. The crowding-out effect has been found to be more significant among resource-constrained low-income households.^14 15^ However, other studies have suggested that the crowding-out effect of tobacco is similar in low-income and high-income groups.^16^

This study aims to estimate the crowding-out effect of tobacco expenditure, both for food and non-food items, among Indonesian households. This contribution is particularly significant since the current evidence in Indonesia is limited to tobacco’s crowding-out effect on food spending.^4 6^ Moreover, this study builds on the latest literature, in which we estimate unbiased estimators by addressing the endogeneity issue using the instrumental variable (IV) approach. Therefore, the finding of this study adds to the literature on the detrimental impact of tobacco spending on household welfare and serves as evidence for stronger tobacco control policies in Indonesia.

### Descriptive statistics

Most Indonesian families are smoking households, with 64% of households in the country buying tobacco products (table 1). The prevalence of tobacco use is higher for low-income and middle-income families (66–67%) compared with the high-income group (52%). This shows that tobacco use is pervasive, as more than half of households buy tobacco products. On average, smoking households spend 407 286 Indonesian rupiah (Rp) on tobacco monthly, which accounts for 10.7% of the total household expenditure. A substantial part of this spending (91%) is spent on kretek (clove cigarettes), while 6% is spent on white cigarettes and the remaining 3% is allocated to other tobacco products. Relative to their income, low-income and middle-income households spend a larger portion of their budget on tobacco compared with the high-income group, with over one-tenth of the spending budget diverted to cigarettes.

**Table 1:** Tobacco expenditure among Indonesian households

Statistics	Overall	Low income	Middle income	High income
The percentage of households that reported tobacco spending	63.72	66.70	66.37	52.45
Average tobacco expenditures (Rp)	407 286	258 290	473 084	619 756
Share of tobacco expenditures out of total spending	10.66%	10.71%	11.30%	8.95%

Source: Pooled Susenas 2017–2019.

US$1=Rp15 700. Expenditures are presented in March 2019 price level. Income groups are determined based on the distribution of households’ per capita expenditures: low income (<41%), middle income (41–80%) and high income (>80%).

Rp, Indonesian rupiah.

As a significant share of the household budget is spent on tobacco, it affects the allocation of household expenditure. Table 2 compares the spending patterns between smoking and non-smoking households. Smoking households spend 49.3% of their total budget on food, which is 1.18 percentage points (pp) lower than non-smoking households. However, on average, smoking families tend to spend a higher budget on staples and beverages than non-smoking families. Meanwhile, on average, smoking households allocate a lower budget for non-food commodities than smoking households, except for alcohol. For instance, smoking households allocate 0.83 and 0.97 pp less on education and healthcare, respectively, relative to non-smoking families. Note that all of the differences presented in table 2 are statistically significant at the 1% level.

**Table 2:** Comparison of share of expenditures between smoking and non-smoking households

Share of total household expenditures (%)	Overall			Difference low income	Difference middle income	Difference high income
	Smoking household	Non-smoking household	Difference			
Food	49.30	50.48	−1.18	−4.86	−2.76	1.19
Staple	10.39	9.74	0.64	−0.60	0.04	0.43
Meat and fish	6.16	6.51	−0.35	−0.72	−0.58	0.11
Dairy	2.72	3.04	−0.31	−0.45	−0.40	−0.14
Fruits and vegetables	6.74	7.42	−0.68	−1.31	−0.98	−0.17
Beverages	5.19	4.63	0.56	0.32	0.49	0.60
Ready-made food	14.17	15.15	−0.98	−1.43	−1.14	0.13
Other food (spices, oils)	3.92	3.98	−0.06	−0.67	−0.19	0.24
Clothing	2.76	2.93	−0.17	−0.14	−0.18	−0.13
Housing	10.03	14.00	−3.97	−2.74	−3.97	−4.10
Utilities and fuel	8.12	10.23	−2.10	−1.70	−1.91	−2.08
Durable and non-durable goods	6.23	7.07	−0.84	−0.50	−0.57	−0.03
Education	2.33	3.15	−0.83	−0.24	−0.65	−1.81
Healthcare	3.33	4.30	−0.97	−0.53	−1.06	−1.26
Transportation	6.08	6.32	−0.24	0.03	−0.11	−0.29
Entertainment	1.12	1.50	−0.39	−0.06	−0.12	−0.49
Alcohol	0.05	0.01	0.04	0.03	0.04	0.05
*Tobacco*	10.66	0.00	10.66	10.71	11.30	8.95
Observations	571 975	336 128		370 685	374 321	163 097

Source: Pooled Susenas 2017–2019.

Differences between smoking and non-smoking households are statistically significant at a 1% level (t-test). Income groups are determined based on the distribution of households’ per capita expenditures: low income (<41%), middle income (41–80%) and high income (>80%).

Overall, smoking households tend to spend less portion of their budget on food and non-food commodities relative to non-smoking families (except for some specific food items such as staples and beverages). This pattern is relatively consistent across income groups (except for some specific food items for high-income households), where the gaps in food expenditures are larger among the low-income groups, while gaps in non-food spending are more pronounced among the top earners. However, this descriptive comparison does not control for household-specific characteristics and other confounders that might explain households’ spending decisions. Failing to do so might lead to biased estimates where researchers inadvertently attribute households’ expenditure allocation to their tobacco spending habits. Therefore, a more credible approach is required to estimate the crowding-out effects of tobacco expenditure.

### Data and method

#### Data

The study uses three rounds of Indonesia’s National Socioeconomic Survey (Susena*s*) from 2017 to 2019. Susenas is a nationally representative survey on household social and economic activities conducted semiannually in March and September. The study uses data from the March survey as it consists of larger respondents compared with the September survey. Susenas collects household expenditure data for over 170 food items and 100 non-food items. Overall, 908 103 households from Susenas 2017–2019 were used for the analysis. Before pooling the surveys, expenditure data were adjusted for inflation using the monthly consumer price index, where all expenditures were presented at March 2019 price level.

#### Theoretical framework

The estimation of the crowding-out effect stems from the underlying assumption that households determine their consumption to maximise a single utility function.^17^ This assumption is relevant as households often pool resources from family members and then make spending decisions based on the needs of their members.^3^ Moreover, a household expenditure survey typically reports spending for the whole family as a single unit. Therefore, the unit analysis in this study is expenditure at the household level.

Household consumption is modelled by a demand function in which the quantity of goods consumed ( $q_{i}$ ) is determined by the price of all goods ( $p_{i}$ ) in the commodities basket, the available budget ( Y ) and the household characteristics ( h ), as outlined in equation 1. Suppose households predetermine the consumption of one good, for example, consumption of tobacco, then they will maximise a utilities function presented in equation 2, where ${\bar{q}}_{n}$ is the predetermined quantity of tobacco consumption, and *M* is the remaining budget after being deducted by the tobacco spending.^18^ Solving equation 2 for *n-1* goods returns a conditional demand function shown in equation 3.

1 $$q_{i}=f^{i}\left(p_{1},p_{2},\ldots ,p_{n};Y;h\right),i=1,2,\ldots n$$

2 $$MaxU=U\left(q_{1},q_{2},\ldots ,{\bar{q}}_{n};a\right),subjectto\sum_{i=1}^{n-1}p_{i}q_{i}=M$$

3 $$q_{i}=g^{i}\left(p_{1},p_{2},\ldots ,p_{n-1},{\bar{q}}_{n};M;h\right),i=1,2,\ldots n-1$$

### Econometric model

As the information on commodity price is unavailable, this study estimates the Engel curve—which allows the use of expenditure data—using the Quadratic Almost Ideal System.^19^ Conditioning on tobacco expenditures, the Engel curves are estimated as follows:

4 $$w_{ij}=(\alpha_{1i}+\alpha_{2i}d_{j}+\alpha_{3ij}p_{nj}{\bar{q}}_{nj}+\delta_{i}^{\prime }h_{j})+(\beta_{1i}+\beta_{2i}{𝑑}_{j})lnM_{j}+(\gamma_{1i}+\gamma_{2i}d_{j})(lnM_{j}{)}^{2}+u_{1i}$$

$w_{ij}$ denotes the share of expenditure of product *i* out of total non-tobacco expenditure for household *j*. Meanwhile, $d_{j}$ is a binary indicator with a value of 1 if the households have a non-zero tobacco expenditure; in other words, $d_{j}$ refers to a smoking household. $p_{nj}{\bar{q}}_{nj}$ is the predetermined tobacco expenditure and $h_{j}$ is a vector for household characteristics, which in this study includes average years of education of adult household members, the share of employed adult members, household composition: number of infants, productive-aged persons, number of seniors in the household, and whether the family lives in a rural or urban area. Lastly, $lnM_{j}$ is the log of total non-tobacco expenditure and ${lnM_{j}}^{2}$ is the square of $lnM_{j}$ .

Tobacco spending ( $p_{nj}{\bar{q}}_{nj}$ ) and the total non-tobacco spending ( $lnM_{j}$ ) in equation 4 are likely to be endogenous due to simultaneity; therefore, an ordinary least square estimation would result in a biased parameter. The latest generation of crowding-out studies addresses this issue with an IV.^8 11–16^ The IV provides consistent estimators if the exogenous instrument is partially correlated with the endogenous regressors (*Cov*[ $z_{i},x_{i}$ ]*≠0*) and the instrument $z_{i}$ only affects the dependent variables $w_{i}$ through the endogenous regressors, or $z_{i}$ does not correlate with the error terms, *E* $\left(u_{i}|z_{i}\right)$ *=0*. As each commodity ( $w_{i}$ ) has the same set of regressors, the study estimates equation 4 by seemingly unrelated regression with the addition of an IV which is effectively a three-stage least square (3SLS) method.^20^

Note that the econometric literature suggests that in the presence of heteroscedastic error, generalized method of moments (GMM) 3SLS estimator is more efficient than the traditional 3SLS.^21^ Unfortunately, our attempt to use GMM 3SLS was unsuccessful as it failed to converge. In addition, it is advisable to report 3SLS with bootstrapped SEs to account for heteroscedasticity.^17^ However, our analysis shows that applying the bootstrap procedure with 1000 replications has an insignificant effect on the SEs and the significance of the estimated parameters, which is likely due to the large number of observations in our data. Moreover, as the bootstrap replication cannot accommodate sample weight in the regression, which is our preferred specification, we decide to estimate equation 4 using the traditional 3SLS regression without bootstrapped SEs.

This study classifies household consumption into 15 commodity groups, which include 7 food items (staples, meat and fish, dairy products, fruits and vegetables, beverages, ready-made food and other food) and 8 non-food items (clothing, housing, utilities and fuel, durable and non-durable goods, education, healthcare, transportation and entertainment). The share of household expenditure on each of these items ( $w_{ij}$ ), which ranges from 0 to 1, is the dependent variable for equation 4 . Alcohol spending is arbitrarily excluded from the 3SLS regression to satisfy the adding-up restriction and because of its negligible share, which only accounts for 0.04% of the total household expenditure.

### Statistical test

A series of statistical tests, which are presented in the online supplemental material, are conducted to ensure the validity of the 3SLS regression. The endogeneity test suggests that endogenous regressors in equation 4, namely tobacco spending, the log of non-tobacco spending and the square of the log of non-tobacco spending, are indeed endogenous for all commodities; therefore, the use of IV is justifiable. Following previous literature, the study employs the log of total expenditures (*lnX*) and its square (*lnX^2^*) as an instrument for the endogenous log of non-tobacco spending (*lnM* and *lnM^2^*). The idea is that households’ economic affluence, as proxied by their total expenditure, only affects their spending structure through expenditure for non-tobacco commodities. Meanwhile, the study employs the share of adult men out of the total adults in the household as the instrument for tobacco expenditure. This is because tobacco consumption in Indonesia is significantly more prevalent among males (65.5%) than females (3.3%).^1^ Therefore, the share of the adult men in the household is highly correlated with tobacco spending. The underidentification test shows a rejection of the null hypothesis, suggesting the proposed instrument is relevant or correlated with the endogenous variables.

10.1136/tc-2022-057843.supp1 Supplementary data

This study also conducts a Wald test for the joint significance of parameters $\alpha_{2i},\beta_{2i},\gamma_{2i}$ , which shows the parameters are jointly different from zero, suggesting a preference heterogeneity between smoking and non-smoking households. In other words, non-smoking households report zero spending on tobacco products because tobacco is not on their utility function, and it is not because tobacco is unaffordable for them. Therefore, equation 4 is the correct regression specification as it accounts for preference heterogeneity.^17^ The other statistical test performed is the heteroscedasticity test which reports heteroscedastic error terms; therefore, heteroscedastic-consistent SEs are employed throughout the analysis.

### Simulation of household spending reallocation

The study includes a simulation to illustrate the change in household expenditure spent on a specific commodity if tobacco spending decreases by 50%. The simulation is calculated based on the following formula:

5 $$\Delta E_{i}=\left(S_{i}^{1}xM^{1}\right)-\left(S_{i}^{0}xM^{0}\right)$$

where $S_{i}^{1}=S_{i}^{0}+\left(\alpha_{3i}x\Delta TobExp\right)$ and $M^{1}=M^{0}-\Delta$ TobExp

${∆E}_{i}$ denotes a change in expenditure for commodity *i* in a monetary unit (Rp). $M^{0}$ is the initial non-tobacco expenditure, while $S_{i}^{0}$ is the share of $M^{0}$ spent on commodity *i*. On the other hand, $S_{i}^{1}$ is the share of expenditure spent on commodity *i* after reduced tobacco spending. Δ TobExp is a negative value as it represents a reduction of tobacco spending by 50% from the initial level. Parameter $\alpha_{3i}$ is the crowding-out coefficient from equation 4. In the case of the crowding-out effect, $\alpha_{3i}$ is negative; hence, the product of $\alpha_{3i}$ and Δ TobExp is a positive value which represents the pp increase in budget share spent on commodity *i* after a reduction in tobacco expenditure. Therefore, in the crowding-out case, $S_{i}^{1}$ is greater than $S_{i}^{0}$ . Lastly, $M^{1}$ refers to the total household’s budget for non-tobacco expenditure after a 50% reduction in tobacco spending. Since Δ TobExp is negative, then $M^{1}$ is greater than $M^{0}$ .

## Result

### The crowding-out effect of tobacco expenditure

Table 3 presents the coefficients of tobacco expenditure from the 3SLS regression, which shows the change in the share of household budget spent on commodities if the tobacco expenditures are increased by Rp100 000—equivalent to buying four packs of the most-sold cigarette brand. Overall, the coefficients show a negative sign and are statistically significant, highlighting that an additional tobacco expenditure reduces the budget share spent on other non-tobacco commodities. For instance, increasing tobacco spending by Rp100 000 will crowd out the share of the remaining budget allotted for staples by 0.0048 pp. Among the food categories, spending on fruits and vegetables is the most negatively impacted as the budget spent on this commodity will decrease by 0.0137 pp for every Rp100 000 addition in tobacco purchase.

**Table 3:** The crowding-out coefficients

	Overall	Low income	Middle income	High income
Food				
Staple	−0.0048***	−0.0077***	−0.001***	−0.0144***
Meat and fish	−0.0094***	0.0007	−0.0029***	−0.0204***
Dairy	−0.0044***	−0.0013***	−0.0016***	−0.0087***
Vegetables and fruits	−0.0137***	−0.0042***	−0.007***	−0.0234***
Beverages	0.0102***	0.0081***	0.0058***	0.0132***
Ready-made food	0.0364***	0.0132***	0.0109***	0.0742***
Other food (spices, oils)	−0.0043***	−0.0013***	−0.0018***	−0.0078***
Clothing	−0.0026***	−0.0008***	−0.0016***	−0.0038***
Housing	−0.0045***	0.0035***	0.0010**	−0.0104***
Utilities and fuels	−0.0018***	−0.0013***	0.0002	−0.0025***
Durable and non-durable goods	−0.0071***	−0.0029***	−0.0045***	−0.0131***
Education	−0.0031***	−0.0042***	−0.0029***	0.0043***
Healthcare	−0.0008***	0.0024***	0.0011***	−0.0011
Transportation	0.0145***	−0.0036***	0.0083***	0.0248***
Entertainment	−0.0049***	−0.001***	−0.0021***	−0.0118***

Source: authors’ estimation based on Susenas 2017–2019 using equation 4.

The table above presents parameters α_3i_, which shows the crowding-out effect of Rp100 000 increase in tobacco expenditure. *** and ** denote significance at 1% and 5% levels, respectively. Extended regression results are available in the online supplemental material. Income groups are determined based on the distribution of households’ per capita expenditures: low income (<41%), middle income (41–80%) and high income (>80%).

Rp, Indonesian rupiah.

For the non-food commodities, spending on entertainment and durable and non-durable goods is the most adversely affected by tobacco expenditure as an additional pre-allocated budget for tobacco by Rp100 000 will reduce the share of expenditure for entertainment (movies, concerts, hotels, religious and social events) by 0.0049 pp and will decrease the share of spending for durable and non-durable goods by 0.0071 pp. At the same time, increasing tobacco spending by the same amount will crowd out the share of expenditure on clothing (0.0026 pp), housing (0.0045 pp), utilities and fuel (0.0018 pp), education (0.0031 pp) and healthcare (0.0008 pp).

On the other hand, additional tobacco expenditure increases the expenditure share for some commodities, such as spending on beverages, ready-made food and transportation. The estimation shows that increasing the pre-allocated spending for cigarettes by Rp100 000 will increase the share of expenditures for beverages (tea, coffee, sugar, bottled drinks) by 0.0102 pp, ready-made food (0.0364 pp) and transportation (0.0145 pp).

The crowding-out analysis disaggregated by household expenditure level reveals that the crowding-out effects of tobacco expenditure persist across income groups, where the effects tend to be higher among the top earners than in the lower-income group. These results are contrary to the expectation that crowding-out effects are more pronounced among low-income households than high-income families. However, our further analysis by splitting the sample by year, modifying the definition of the income groups and using an alternative instrument variable for tobacco spending produced relatively consistent findings that the crowding effect of tobacco tends to be higher among high-income households.

One of the possible reasons explaining this result is the heterogeneity of consumption behaviour across income groups, where tobacco spending tends to induce consumption of other commodities more profoundly for a specific income group. For example, an increase in tobacco expenditure has a higher impact on increasing the budget share spent on beverages, ready-made food and transportation among high-income households than middle-income or low-income households (see table 3). This consequently leads to a higher crowding-out effect among the high-income group, as they have to compensate for a higher budget share spent on beverages and ready-made food in addition to increased tobacco expenditure.

Tobacco spending consistently reduces the budget share spent on staples, dairy products, vegetables and other food across the income groups and decreases spending on meat for middle-income and high-income households. On the other hand, tobacco spending is consistently associated with an increased household budget share allotted for beverages and ready-made food across all income groups. In addition, tobacco spending also crowds out the budget share allocated for durable and non-durable goods, clothing and entertainment across income levels. Moreover, the analysis finds that increased tobacco spending reduces the budget share spent on housing among middle-income and high-income groups. Meanwhile, the crowding-out effect of tobacco spending on education only occurs among low-income and middle-income families, while the same effect on healthcare spending is only found among top-income households.

The study conducts a robustness check by employing alternative instrument variables for tobacco expenditure. Following previous studies,^4 13 15 17^ we use a composite measure of smoking prevalence, which is constructed using other independent datasets, as an alternative instrument for tobacco spending.^12 14^ The household’s smoking prevalence is predicted using the 2017 Indonesia Demographic Health Survey. The results of the robustness check are available in the online supplemental material. Overall, the results show that crowding-out effects are consistent across all proposed instruments. Moreover, the robustness check also confirms the main finding that tobacco expenditure is positively associated with spending on beverages, ready-made food and transportation.

### The simulation of tobacco expenditure reduction by 50%

The study simulates changes in the household’s expenditure if tobacco spending is reduced by 50%, which assumes that saving from reduced tobacco spending is fully reallocated to other non-tobacco commodities. Table 4 shows the average monthly expenditure among smoking households is Rp4 259 947, of which Rp407 283 is spent on tobacco and Rp3 852 662 is allocated to other non-tobacco commodities. A 50% reduction means that tobacco spending decreases by Rp203 643, which increases the budget available for non-tobacco commodities by the same amount from Rp3 852 662 to Rp4 056 305. Note that the simulations in table 4 are calculated using equation 5 and are based on the crowding-out coefficients for the overall populations.

**Table 4:** Simulation of the impact of a 50% tobacco spending reduction on household expenditure

Total household expenditure (Rp)		4 259 947	
Total non-tobacco expenditure (Rp)		3 852 662	
Tobacco expenditure (Rp)		407 285	
A decrease in tobacco expenditure by 50% (Rp)		−203 643	
	**Initial expenditure (Rp**)	**Additional expenditure due to reduction in tobacco expenditure (Rp**)	**Percentage increase from the initial expenditure**
Food			
Staple	450 806	63 065	14.0
Meat and fish	266 099	92 043	34.6
Dairy	116 997	42 447	36.3
Vegetable and fruit	291 185	128 558	44.2
Beverage	225 390	−72 342	−32.1
Ready-made food	612 019	−268 327	−43.8
Other food (spices, oils)	170 199	44 681	26.3
Clothing	119 263	27 864	23.4
Housing	432 458	60 278	13.9
Utilities and fuels	350 322	33 138	9.5
Durable and non-durable goods	266 056	73 042	27.5
Education	98 610	30 654	31.1
Healthcare	142 068	14 184	10.0
Transportation	262 047	−105 924	−40.4
Entertainment	46 917	43 286	92.3

Source: authors’ estimation based on equation 5.

US$1=Rp15 700. The average expenditures presented in rows 1–4 and the initial expenditure are statistics among smoking households derived from Susenas 2017–2019. The figures have been adjusted for inflation and are presented in March 2019 price level.

Rp, Indonesian rupiah.

Assuming everything else is constant, the simulation shows that reducing tobacco spending will increase spending on food items, except for beverages and ready-made food. Reducing tobacco expenditure by 50% from the current level will increase spending on staples by Rp63 065, or 14% higher than the current expenditure. In addition, it also increases spending on fruits and vegetables (44.2%), dairy products (36.3%), meat and fish (34.6%) and other food (26.3%). On the other hand, spending less on tobacco by the same amount will reduce expenditure on ready-made food by 43.8% and on beverages by 32.1%. Cutting cigarette spending by half also increases expenditure on education (31.1%), durable and non-durable goods (27.5%), housing (13.9%), healthcare (10%) and utilities (9.5%). On the contrary, it will reduce the budget allocated for transportation by 40.4%.

## Discussion and conclusion

Tobacco consumption is pervasive in Indonesia, with at least one smoker in 6 out of 10 households reporting spending on tobacco products. Smoking households divert a significant share of their budget on tobacco, where around 10.7% of the monthly expenditure is spent on cigarettes and other tobacco products. This study aims to estimate the crowding-out effects of tobacco spending on the consumption of other goods and services. Using the annual Indonesia socioeconomic survey (Susenas) from 2017 to 2019, comprising over 908 103 households, this study analyses the crowding-out effect by estimating the conditional Engel curve with 3SLS to account for the endogeneity of tobacco expenditure.

The descriptive analysis suggests that smoking households, on average, allocate a lower portion of their spending on non-tobacco commodities compared with non-smoking households (except for spending on alcohol), and the gap is more prominent among low-income earners. The crowding-out analysis confirms that additional tobacco spending reduces the percentage of expenditure allocated for food, such as staples, meat, dairy, fruits and vegetables, and spending on other food (spices and oils). These findings are consistent with evidence from previous studies in Indonesia which suggested that higher tobacco spending leads to reduced food expenditure and poorer diet quality.^4 6^ Moreover, the findings in this study are in agreement with evidence from other countries such as India, Cambodia, Turkey and Serbia, suggesting that tobacco spending crowds out expenditure on food items.^8 11 14 22^ In addition to diverting spending on food, the research also finds that tobacco spending crowds out the share of expenditure spent on non-food commodities, although its effect is not as large as the crowding out on food. The estimate shows that additional tobacco spending would reduce resources allocated to clothing, housing, utilities, durable and non-durable goods, education, healthcare and entertainment.

The tobacco’s crowding-out effect on food consumption found in this study helps to explain nutritional inadequacy among the smoking families documented in previous studies. Previous studies have documented that poor smokers in Indonesia diverted a substantial portion of an already limited resource to tobacco, reducing the dietary quantity and quality.^4^ Consequently, individuals living in a smoking household in the country have lower protein intake than those living in non-smoking families, where the gap in nutritional adequacy is more significant among low-income smokers.^5^ Therefore, this study’s findings serve as credible evidence that tobacco spending crowds out resources allocated to food and directly contributes to poor diets and nutrition inadequacy among smoking families in Indonesia.

The findings of this study also demonstrate that tobacco spending is positively associated with the budget share allocated to beverages and ready-made food, as well as transportation. The possible explanation for the close association between cigarette smoking and health-compromising behaviour (including consumption of caffeinated and sweetened beverages) is highlighted in the literature as being mediated through several mechanisms, including physiological and psychological mechanisms.^23–25^ Additionally, a recent body of evidence reveals that cigarette smoking is strongly associated with poor dietary choices, such as a more frequent intake of energy-dense food and a lower intake of nutritious food.^26–29^ As for the positive link between cigarette expenditure and transportation, though the evidence is still inconclusive in the literature,^10 30–33^ some studies explain that this might stem from the potential correlation between cigarette smoking and social activities.^32 33^ Besides, in some areas where cigarette is not accessible, higher spending on transportation in the smoking household might possibly be linked with more budget for transportation fares.^25 28^

Reduced spending on food and other essential commodities such as housing, education and healthcare among smoking families might detrimentally affect human capital investment over the long run, particularly for children. A recent estimate shows that children with smoking parents in Indonesia have increased odds of stunting and lower growth indices.^7 34^ Moreover, it has been found that Indonesian children growing up in smoking households have lower cognitive scores than those living in non-smoking families.^35^ This indicates that the crowding-out effect of tobacco potentially brings a long-term and intergenerational adverse impact as it reduces children’s productivity and earnings in adulthood.^36 37^

One of the limitations of this study is that we are unable to look at the intrahousehold resource allocation due to the crowding-out effect of tobacco expenditure. For example, the reduced budget share allocated to certain commodities might disproportionately affect specific family members. Nevertheless, due to data availability, analysis of individual-level consumption is not possible. Moreover, this study uses the traditional 3SLS, which produces less efficient estimators than GMM 3SLS. Finally, our finding on the differential of the crowding-out effect across income groups differs from previous literature, where the crowding-out effect of tobacco expenditure is typically higher among low-income households.^14 15^ Nevertheless, a battery of robustness checks confirms the consistency of our findings that the crowding-out effect of tobacco spending in Indonesia tends to be larger among high-income households relative to lower ones.

In conclusion, this research demonstrates that tobacco spending in Indonesia reduces the budget allocated to food and non-food commodities, where the crowding-out effect is higher for food items than non-food items. Moreover, the analysis suggests that the crowding-out effect persists across the income groups. Given that more than half of Indonesian families are smoking households, the crowding-out effect of tobacco affects a significant share of the population, including those who do not smoke. As high tobacco spending in Indonesia adversely crowds out expenditure on basic commodities, this study highlights the need for a more effective tobacco control policy that supports a reduction of tobacco consumption and tobacco expenditure in the country. Reduced tobacco expenditure would improve the welfare of the smoking household as it would free up resources for essential needs such as food, housing, education and healthcare, which is essential for human capital investment.

## Data Availability

Data may be obtained from a third party and are not publicly available. The data are procured from Statistic Indonesia, and they cannot be shared with third parties due to copyright protection.
