# Supplementary material for: Crowding-out effect of tobacco consumption in Indonesia
Source: Tob Control. 2024 Jan 22;33(Suppl 2):s81–7. doi: 10.1136/tc-2022-057843 (PMC11187382; doi:10.1136/tc-2022-057843)
Supplement: Supplementary data [file tc-2022-057843supp001.pdf]

SUPPLEMENTARY MATERIAL

Table A1. Descriptive statistics of variables included in the crowding-out estimation

Variable name	Variable label	All sample (n=908,103)		Low income (n=370,685)		Middle income (n=374,321)		High income (n=163,097)	
		Mean	Std dev	Mean	Std dev	Mean	Std dev	Mean	Std dev
**Dependent variables**									
Staple	Share of staple food exp. out of total non-tobacco expenditures	0.1099	0.0714	0.1603	0.0713	0.0931	0.0456	0.0427	0.0320
Meat and fish	Share of meat and fish exp. out of total non-tobacco expenditures	0.0676	0.0500	0.0685	0.0506	0.0731	0.0497	0.0548	0.0472
Dairy	Share of dairy food exp. out of total non-tobacco expenditures	0.0304	0.0321	0.0313	0.0302	0.0317	0.0334	0.0259	0.0327
Fruit and vegetables	Share of vegetable and fruit exp. out of total non-tobacco expenditures	0.07509	0.04085	0.08485	0.04009	0.07632	0.03905	0.05308	0.03743
Beverages	Share of beverages exp. out of total non-tobacco expenditures	0.0541	0.0306	0.0599	0.0308	0.0538	0.0295	0.0431	0.0291
Ready-made food	Share of ready-made food exp. out of total non-tobacco expenditures	0.1562	0.0992	0.1521	0.0918	0.1605	0.0996	0.1557	0.1116
Other food	Share of other food (spices, oils) exp. out of total non-tobacco expenditures	0.0426	0.0243	0.0537	0.0238	0.0412	0.0209	0.0231	0.0174
Clothing	Share of clothing exp. out of total non-tobacco expenditures	0.0304	0.0201	0.0275	0.0170	0.0325	0.0204	0.0317	0.0240
Housing	Share of housing exp. out of total non-tobacco expenditures	0.1223	0.0832	0.0966	0.0611	0.1282	0.0777	0.1622	0.1099
Utilities and fuels	Share of utilities exp. out of total non-tobacco expenditures	0.0951	0.0498	0.0873	0.0387	0.0927	0.0436	0.1152	0.0713
Durable and non-durable goods	Share of durable & non-durable goods exp. out of total non-tobacco expenditures	0.0697	0.0764	0.0503	0.0384	0.0721	0.0671	0.1037	0.1238
Education	Share of education exp. out of total non-tobacco expenditures	0.0278	0.0443	0.0256	0.0317	0.0263	0.0401	0.0350	0.0674
Health care	Share of health care exp. out of total non-tobacco expenditures	0.0391	0.0563	0.0361	0.0414	0.0388	0.0557	0.0456	0.0785
Transportation	Share of transportation exp. out of total non-tobacco expenditures	0.0663	0.0520	0.0599	0.0496	0.0671	0.0484	0.0773	0.0611
Entertainment	Share of entertainment exp. out of total non-tobacco expenditures	0.0132	0.0510	0.0057	0.0212	0.0120	0.0425	0.0306	0.0901

Variable name	Variable label	All sample (n=908,103)		Low income (n=370,685)		Middle income (n=374,321)		High income (n=163,097)	
		Mean	Std dev	Mean	Std dev	Mean	Std dev	Mean	Std dev
Alcohol	Share of alcohol exp. out of total non-tobacco expenditures	0.0004	0.0057	0.0004	0.0048	0.0004	0.0057	0.0004	0.0073
**Endogenous variables**									
exptob	Total amount of tobacco expenditure (Rp)	259,522	342,563	172,289	212,150	313,999	359,550	325,034	461,235
lnM	Log of total non-tobacco expenditure	14.92	0.72	14.41	0.51	15.05	0.49	15.71	0.66
lnM^2	Square of (log) total non-tobacco expenditure	223.25	21.74	207.82	14.43	226.63	14.65	247.39	20.89
**Preference heterogeneity variables**									
tob	Dummy variable for tobacco spender	0.64	0.48	0.67	0.47	0.66	0.47	0.52	0.50
tob x lnM	Interaction term	9.51	7.20	9.65	6.83	10.01	7.13	8.24	7.86
$(tob \times lnM)^2$	Interaction term	142.30	108.52	139.87	99.37	151.10	108.10	129.58	124.22
**Instrument variables**									
lnX	Total household expenditure (log)	14.9981	0.7177	14.4846	0.5181	15.1278	0.4870	15.7655	0.6436
lnX^2	Total household expenditure (log, squared)	225.4570	21.5977	210.0719	14.8176	229.0887	14.6020	248.9647	20.3562
madultshare	Share of male adult out of total household member	0.4883	0.2112	0.4742	0.1853	0.4912	0.2027	0.5108	0.2670
**Alternative instrument variables**									
madultratio	Ratio of male adult to female adult household member	1.0803	0.7084	1.0792	0.7053	1.1055	0.7129	1.0319	0.7029
predictsmoke_dhs17	Predicted probability of smoking (household member average)<br>based on smoking determinant parameters obtained from DHS17	0.3606	0.1511	0.3773	0.1381	0.3577	0.1450	0.3329	0.1807
predictsmoke_sus20	Predicted probability of smoking (household member average)<br>based on smoking determinant parameters obtained from Susenas 2020	0.2904	0.2319	0.2846	0.2093	0.3023	0.2285	0.2779	0.2761
**Control variables**									
yeduc	Average years of education of adult household members	7.8510	3.3347	7.0345	2.8532	8.0716	3.1336	9.0427	4.1024
hhsize	Number of household members	3.7592	1.6516	4.2176	1.7028	3.6489	1.5065	3.0628	1.5400
nchild05	Number of children 0 to 5 years old in the household	0.4028	0.6129	0.5230	0.6692	0.3641	0.5768	0.2395	0.5084
nchild15	Number of children 6 to 14 years old in the household	0.6033	0.7681	0.7731	0.8410	0.5548	0.7135	0.3606	0.6295
nsenior65	Number of seniors >65 years old in the household	0.1961	0.4693	0.2580	0.5322	0.1650	0.4275	0.1343	0.3947
sworking	Share of adult household member who work	0.5860	0.2884	0.5571	0.2699	0.5962	0.2819	0.6231	0.3285

Variable name	Variable label	All sample (n=908,103)		Low income (n=370,685)		Middle income (n=374,321)		High income (n=163,097)	
		Mean	Std dev	Mean	Std dev	Mean	Std dev	Mean	Std dev
urban	1 if household live in urban area	0.5462	0.4979	0.4279	0.4948	0.5293	0.4991	0.8166	0.3870
y17	=1 if year=2017 (base)	0.3287	0.4697	0.3287	0.4697	0.3287	0.4698	0.3287	0.4697
y18	=1 if year=2018	0.3325	0.4711	0.3325	0.4711	0.3325	0.4711	0.3325	0.4711
y19	=1 if year=2019	0.3388	0.4733	0.3388	0.4733	0.3388	0.4733	0.3388	0.4733

Source: Pooled Susenas (2017-2019)

Notes: Expenditure data have been adjusted for inflation and are presented in March 2019 price level. Income groups are determined based on the distribution of households' per capita expenditures: Low-income (<41%), Middle-income (41%-80%), and High-income (>80%).

Table A2. Statistical Test (All households)

	(1)	(2)	(3)	(4)	(5)	(6)	(7)	(8)	(9)	(10)	(11)	(12)	(13)	(14)	(15)
	Staple	Meat and fish	Dairy	Fruit and vegetables	Beverages	Ready-made food	Other food	Clothing	Housing	Utilities and fuels	Durable & non-durable goods	Education	Health-care	Transpor-tation	Entertain-ment
**Heteroskedasticity test**	31286.01	1809.58	18752.75	163.57	489.23	734.20	1558.57	2137.63	19708.59	19685.56	68816.97	9469.33	7334.00	1437.80	20572.26
(Pagan-Hall general test statistic, p-value)	(0.0000)	(0.0000)	(0.0000)	(0.0000)	(0.0000)	(0.0000)	(0.0000)	(0.0000)	(0.0000)	(0.0000)	(0.0000)	(0.0000)	(0.0000)	(0.0000)	(0.0000)
**Under identification test**	4609.09	4609.09	4609.09	4609.09	4609.09	4609.09	4609.09	4609.09	4609.09	4609.09	4609.09	4609.09	4609.09	4609.09	4609.09
(Kleibergen-Paap rk LM-test, p-value)	(0.0000)	(0.0000)	(0.0000)	(0.0000)	(0.0000)	(0.0000)	(0.0000)	(0.0000)	(0.0000)	(0.0000)	(0.0000)	(0.0000)	(0.0000)	(0.0000)	(0.0000)
**Weak identification test** (Kleibergen-Paap rk Wald F statistic)	1580.87	1580.87	1580.87	1580.87	1580.87	1580.87	1580.87	1580.87	1580.87	1580.87	1580.87	1580.87	1580.87	1580.87	1580.87
**Endogeneity test**	408.74	1187.66	748.56	3256.49	2440.81	1856.51	976.09	843.48	292.13	257.75	295.81	142.02	30.40	2195.41	292.61
(GMM-C-Statistics, p-value)	(0.0000)	(0.0000)	(0.0000)	(0.0000)	(0.0000)	(0.0000)	(0.0000)	(0.0000)	(0.0000)	(0.0000)	(0.0000)	(0.0000)	(0.0000)	(0.0000)	(0.0000)
**Household preference test**	4604.45	1302.70	856.45	2193.69	3485.66	1858.62	1105.92	534.70	550.07	168.38	433.09	117.62	35.24	2328.25	258.92
(Chi-square, p-value)	(0.0000)	(0.0000)	(0.0000)	(0.0000)	(0.0000)	(0.0000)	(0.0000)	(0.0000)	(0.0000)	(0.0000)	(0.0000)	(0.0000)	(0.0000)	(0.0000)	(0.0000)

Notes: Results of statistical test by income groups are available upon request

Table A3. Result of 3SLS regression (All households)

	(1)	(2)	(3)	(4)	(5)	(6)	(7)	(8)
**All group**	**Staple**	**Meat and fish**	**Dairy**	**Fruit and vegetables**	**Beverages**	**Ready-made food**	**Other food (spices, oils)**	**Clothing**
Dummy variable for tobacco spender (***tob***)	0.712***	-1.335***	-0.407***	-1.499***	0.786***	0.901***	-0.495***	-0.262***
	(0.0331)	(0.0371)	(0.0217)	(0.0344)	(0.0250)	(0.0878)	(0.0158)	(0.0142)
Total amount of tobacco spending (***exptob***)	-0.0048***	-0.0094***	-0.0044***	-0.0137***	0.0102***	0.0364***	-0.0043***	-0.0026***
	(0.00002)	(0.00002)	(0.00001)	(0.00002)	(0.00002)	(0.00005)	(0.00001)	(0.00001)
Log of total non-tobacco expenditure (***lnM***)	-0.279***	0.144***	0.0284***	-0.00943***	0.0866***	0.343***	-0.0334***	0.0396***
	(0.00265)	(0.00297)	(0.00173)	(0.00276)	(0.00200)	(0.00703)	(0.00126)	(0.00114)
Square of (log) total non-tob expenditure (***lnM)***^{2}	0.00742***	-0.00465***	-0.0009***	-0.00001	-0.00335***	-0.0127***	0.000639***	-0.00114***
	(0.00009)	(0.0001)	(0.00006)	(0.00009)	(0.00007)	(0.00024)	(0.00004)	(0.00004)
Interaction term (***tob x lnM***)	-0.0864***	0.163***	0.0471***	0.180***	-0.0874***	-0.0881***	0.0613***	0.0310***
	(0.00429)	(0.00481)	(0.00281)	(0.00446)	(0.00324)	(0.0114)	(0.00204)	(0.00184)
Interaction term [(***tob x lnM***)^2]	0.00270***	-0.00475***	-0.00125***	-0.00506***	0.00218***	0.00124***	-0.00180***	-0.000851***
	(0.000137)	(0.000154)	(0.00009)	(0.000143)	(0.000104)	(0.000365)	(0.00007)	(0.00006)
Household characteristics	Yes	Yes	Yes	Yes	Yes	Yes	Yes	Yes
Year dummy	Yes	Yes	Yes	Yes	Yes	Yes	Yes	Yes
Observations	908,103	908,103	908,103	908,103	908,103	908,103	908,103	908,103
R-squared	0.529	-0.211	-0.002	-0.562	-0.469	-0.725	0.072	-0.103

Source: Authors' estimation based on Susenas 2017-2019 using Equation 4

Notes: Standard error is reported in parentheses. Parameters *exptob* are multiplied by 100,000. *** , ** , and * denote significance at 1%, 5%, and 10% levels, respectively. Parameters of household characteristics and year dummy are not reported in this table. The household's characteristics include whether they lived in a rural/urban area, average years of education of adult household members, the share of adult members who work, household composition: number of infants, productive age persons, and seniors in the household. Regression results by income groups are available upon request.

Table A3. Result of 3SLS regression (All households) - continued

	(9)	(10)	(11)	(12)	(13)	(14)	(15)
**All group**	**Housing**	**Utilities and fuels**	**Durable and non-durable goods**	**Education**	**Health care**	**Transportation**	**Entertainment**
Dummy variable for tobacco spender (***tob***)	-0.835***	0.247***	0.585***	-0.00525	0.235***	1.440***	-0.0918***
	(0.0516)	(0.0322)	(0.0491)	(0.0274)	(0.0373)	(0.0415)	(0.0343)
Total amount of tobacco spending (***exptob***)	-0.0045***	-0.0018***	-0.0071***	-0.0031***	-0.0008***	0.0145***	-0.0049***
	(0.00003)	(0.00002)	(0.00003)	(0.00002)	(0.00002)	(0.00003)	(0.00002)
Log of total non-tobacco expenditure (***lnM***)	-0.0339***	-0.209***	-0.214***	0.00244	0.0201***	0.217***	-0.105***
	(0.00413)	(0.00258)	(0.00393)	(0.00219)	(0.00299)	(0.00333)	(0.00274)
Square of (log) total non-tob expenditure ($\boldsymbol{lnM})^2$	0.00183***	0.00745***	0.00843***	0.000227***	-0.000305***	-0.00701***	0.00414***
	(0.00014)	(0.00009)	(0.00013)	(0.00007)	(0.0001)	(0.00011)	(0.00009)
Interaction term (***tob x lnM***)	0.101***	-0.0303***	-0.0936***	0.000242	-0.0324***	-0.168***	0.00545
	(0.00668)	(0.00417)	(0.00636)	(0.00355)	(0.00484)	(0.00538)	(0.00444)
Interaction term [(***tob x lnM***)^2]	-0.00296***	0.000921***	0.00378***	0.00003	0.00111***	0.00452***	0.000132
	(0.000214)	(0.000134)	(0.000204)	(0.000114)	(0.000155)	(0.000173)	(0.000142)
Household characteristics	Yes	Yes	Yes	Yes	Yes	Yes	Yes
Year dummy	Yes	Yes	Yes	Yes	Yes	Yes	Yes
Observations	908,103	908,103	908,103	908,103	908,103	908,103	908,103
R-squared	0.155	0.081	0.093	0.160	0.034	-0.402	0.010

Source: Authors' estimation based on Susenas 2017-2019 using Equation 4

Notes: Standard error is reported in parentheses. Parameters *exptob* are multiplied by 100,000. *** , ** , and * denote significance at 1%, 5%, and 10% levels, respectively. Parameters of household characteristics and year dummy are not reported in this table. The household's characteristics include whether they lived in a rural/urban area, average years of education of adult household members, the share of adult members who work, household composition: number of infants, productive age persons, and seniors in the household. Regression results by income groups are available upon request.

Table A4. The crowding-out effect of tobacco expenditures using alternative instruments

	**Main instrument**	**Instrument alternative**
	male adult share lnX, lnX^2	predicted smoke from DHS17, lnX, lnX^2
Food		
Staple	-0.0048***	-0.0015***
Meat and fish	-0.0094***	-0.007***
Dairy	-0.0044***	-0.0037***
Fruit and vegetables	-0.0137***	-0.0086***
Beverages	0.0102***	0.0088***
Ready-made food	0.0364***	0.0294***
Other food (spices, oils)	-0.0043***	-0.0022***
Clothing	-0.0026***	-0.0025***
Housing	-0.0045***	-0.0048***
Utilities and fuels	-0.0018***	-0.0049***
Durable and non-durable goods	-0.0071***	-0.0019***
Education	-0.0031***	-0.0076***
Health care	-0.0008***	-0.0012***
Transportation	0.0145***	0.01***
Entertainment	-0.0049***	-0.0027***

Source: Authors' estimation based on Susenas 2017-2019 using Equation 4
Notes: The table above presents parameters exptob, multiplied by 100,000. ***, **, and * denote significance at 1%, 5%, and 10% levels, respectively. Survey weight is applied in the regression. There are different instrument variables used in each alternative. Main instrument: share of adult males out of adult household members (madultshare), log of total expenditure (lnX) and its square (lnX2). Alternative instrument: Predicted probability of smoking using parameters from Demographic Health Survey 2017 (predictedsmoke_dhs17), (lnX), and (lnX2).
